# Supplementary material for: Public health benefits of shifting from hospital-focused to ambulatory TB care in Eastern Europe: Optimising TB investments in Belarus, the Republic of Moldova, and Romania
Source: PLOS Glob Public Health. 2023 Jun 21;3(6):e0001025. doi: 10.1371/journal.pgph.0001025 (PMC10284374; doi:10.1371/journal.pgph.0001025)
Supplement: S1 Text — (DOCX) [file pgph.0001025.s001.docx]

Supporting Information: S1 Text

**Public health benefits of shifting from hospital-focused to ambulatory TB care in Eastern Europe: optimising TB investments in Belarus, the Republic of Moldova, and Romania**

Sherrie L Kelly,^1^ Gerard Joseph Abou Jaoude,^2^ Tom Palmer,^2^ Jolene Skordis,^2^ Hassan Haghparast-Bidgoli,^2^ Lara Goscé,^2^ Sarah J Jarvis,^3^ David J Kedziora,^4^ Romesh Abeysuriya,^1^ Clemens Benedikt,^5^ Nicole Fraser-Hurt,^5^ Zara Shubber,^5^ Nejma Cheikh,^5^ Stela Bivol,^6^ Anna Roberts,^1^ David P Wilson,^1^ Rowan Martin-Hughes^1^

^1^Burnet Institute, Melbourne, Australia

^2^Institute for Global Health, University College London, London, UK

^3^Applied Sciences, Babylon Health, London, UK

^4^Complex Adaptive Systems Lab, University of Technology Sydney, Sydney, Australia

^5^World Bank Group, Washington, DC, USA

^6^World Health Organization, Regional Office for Europe, Copenhagen, Denmark

This Supporting Information File provides additional details on the Optima TB analysis conducted for Belarus, the Republic of Moldova, and Romania. This document accompanies the manuscript *Public health benefits of shifting from hospital-focused to ambulatory TB care in Eastern Europe: optimising TB investment in Belarus, the Republic of Moldova, and Romania.*

Hospital-focused and ambulatory durations for different TB treatment modalities used to inform the three Optima TB model country analyses are shown in Table A for Belarus, Table C for the Republic of Moldova, and Table D for Romania. Costs associated with these TB treatment modalities are in 2015 US dollars in Table B for Belarus, 2016 USD in Table C for Moldova, and 2016 USD in Table E for Romania.

Table A summarises the durations of TB treatment, which were assumed for the different treatment modalities based on WHO recommendations and consultation with in-country experts. The assumed reduction in hospital-focused days for ambulatory modalities corresponds to the estimated time required to achieve smear conversion suggesting that the patient is no longer highly infectious. Available data suggest that current average duration of hospitalisation in Belarus is substantially longer than required on average to achieve smear conversion.

Table A. Duration of hospital-focused and ambulatory TB treatment by modality for Belarus from the 2016−2017 study [1]

| **Indicator** | **Treatment type** | **Hospital-focused (current)** | **Standard ambulatory**^1^ | **Incentivised ambulatory**^2^ |
| --- | --- | --- | --- | --- |
| Total days | DS | 180 | 180 | 180 |
|  | MDR long-course^3^ | 600 | 600 | 600 |
|  | MDR short-course^4^ | Not applicable | 315 | 315 |
|  | XDR | 720 | 720 | 720 |
| Number of ambulatory days | DS | 120 | 166 | 166 |
|  | MDR long-course^3^ | 390 | 555 | 555 |
|  | MDR short-course^4^ | Not applicable | 285 | 285 |
|  | XDR | 450 | 660 | 660 |
| Number of hospital-focused days | DS | 60 | 14 | 14 |
|  | MDR long-course^3^ | 210 | 45 | 45 |
|  | MDR short-course^2^ | Not applicable | 30 | 30 |
|  | XDR | 270 | 60 | 60 |
| Relative increase in treatment success rate | All (DS, MDR long-course,^3^ MDR short-course,^4^ XDR) | Standard (baseline) | No change | 16% (through reduced loss-to follow-up) |

^1^Standard ambulatory indicates that financial incentives were not incorporated.

^2^Incentivised ambulatory indicates that financial incentives were incorporated.

^3^MDR long-course is the longer MDR regimen with treatment up to 18-24 months.

^4^MDR short-course is the shorter MDR regimen with a four month intensive phase (extended to six months in case of delayed sputum smear conversion) containing high-dose gatifloxacin or moxifloxacin, kanamycin, prothionamide, clofazimine, high-dose isoniazid, pyrazinamide and ethambutol followed by a continuation phase of five months containing gatifloxacin or moxifloxacin, clofazimine, ethambutol and pyrazinamide.

DS=drug-susceptible. MDR=multidrug-resistant. MDR plus=treatment with re-purposed medications such as Linezolid (LNZ), Imepinem/Cilastatin, and Amoxicillin/Clavulanic acid. XDR=extensively drug-resistant. Values were provided by national experts as part of the 2016−2017 study [1].

Table B provides a breakdown of the estimated cost for different treatment modalities for Belarus based on a combination of estimated drug costs, duration of hospitalisation and associated costs, and costs for any incentives to transition to ambulatory care. The costs in the table represent expenses for a long-course of TB treatment for one patient for different treatment modalities. A description of assumptions used to inform these costs can be found in Appendix C of the Belarus investment case report [1]. A shift to ambulatory treatment modalities would reduce the cost per patient on treatment by 25−28% for DS, MDR, and XDR-TB. Even if the cost to offer incentives for health workers to provide ambulatory services were included in these costs, it is estimated that costs could be reduced by 15−20% per patient. The largest saving could be realised if involuntary isolation treatment were to be reduced.

Table B. Estimated cost of a full course of treatment for different drug regimens by treatment modality for Belarus from the 2016−2017 study (costs given in 2015 USD) [1]

| Regimen | Hospital-focused  (USD) | Standard ambulatory  (USD) | Incentivised ambulatory^1^  (USD) | Involuntary isolation  (USD) |
| --- | --- | --- | --- | --- |
| DS | 2,610 | 1,878 | 2,215 | Not applicable |
| MDR short-course^2^ | Not applicable | 4,520 | 5,100 | Not applicable |
| MDR long-course^3^ | 14,158 | 10,196 | 11,325 | 21,482 |
| XDR | 20,483 | 15,441 | 16,783 | 28,840 |

^1^Incentivised ambulatory indicates that financial incentives were incorporated.

^2^MDR short-course is the shorter MDR regimen with a four month intensive phase (extended to six months in case of delayed sputum smear conversion) containing high-dose gatifloxacin or moxifloxacin, kanamycin, prothionamide, clofazimine, high-dose isoniazid, pyrazinamide and ethambutol followed by a continuation phase of five months containing gatifloxacin or moxifloxacin, clofazimine, ethambutol and pyrazinamide.

^3^MDR long-course is the longer MDR regimen with treatment up to 18-24 months.

DS=drug-susceptible. MDR=multidrug-resistant. MDR plus=treatment with re-purposed medications such as Linezolid (LNZ), Imepinem/Cilastatin, and Amoxicillin/Clavulanic acid. XDR=extensively drug-resistant.

Table C summarises the durations and costs of TB treatment for different modalities for Moldova. The assumed reduction in hospital hospital-focused days when services are delivered using ambulatory modalities corresponds to the estimated time required to achieve smear conversion, suggesting that the patient is no longer highly infectious.

**Table C. Duration and cost of hospital-focused and ambulatory TB treatment modalities for Moldova from the 2017−2018 study (costs given in 2016 USD) [2]**

| **Program** | **Treatment regimen** | **Length of treatment duration**  **(days)** | **Drug regimen cost, (full course (USD)** | **Hospital-focused** | |  | **Ambulatory/DOTS** | |
| --- | --- | --- | --- | --- | --- | --- | --- | --- |
|  |  |  |  | **Days** | **Costs**  **(USD)** |  | **Days** | **Costs**  **(USD)** |
| Hospital-focused (current) | DS | 201 | 28.70 | 40 | 835.18 |  | 161 | 525.56 |
|  | MDR standard | 570 | 1,826.15 | 127 | 2,621.58 |  | 444 | 1,501.00 |
|  | MDR plus | 570 | 2,637.48 | 127 | 2,621.58 |  | 444 | 1,501.00 |
|  | Pre-XDR | 720 | 2,637.48 | 160 | 3,311.47 |  | 560 | 1,896.00 |
|  | Pre-XDR new drugs | 570 | 5,874.00 | 127 | 2,621.58 |  | 444 | 1,501.00 |
|  | XDR | 880 | 2,637.48 | 195 | 4,047.35 |  | 685 | 2,317.34 |
|  | XDR new drugs | 720 | 8,658.51 | 160 | 3,311.47 |  | 560 | 1,896.00 |
| Hospital-focused (reduced hospitalisation) | DS | 201 | 28.70 | 14 | 290.14 |  | 187 | 611.57 |
|  | MDR standard | 570 | 1,826.15 | 60 | 1,243.44 |  | 510 | 1,726.07 |
|  | MDR-plus | 570 | 2,637.48 | 60 | 1,243.44 |  | 510 | 1,726.07 |
|  | Pre-XDR | 720 | 2,637.48 | 60 | 1,243.44 |  | 660 | 2,233.74 |
|  | Pre-XDR new drugs | 570 | 5,874.00 | 60 | 1,243.44 |  | 510 | 1,726.07 |
|  | XDR | 880 | 2,637.48 | 60 | 1,243.44 |  | 820 | 2,775.25 |
|  | XDR new drugs | 720 | 8,658.51 | 60 | 1,243.44 |  | 660 | 2,233.74 |

DOTS=directly observed treatment, short-course. DS=drug-susceptible. MDR=multidrug-resistant. MDR plus=treatment with re-purposed medications such as Linezolid (LNZ), IMEPINEM/Cilastatin, and Amoxicillin/Clavulanic acid. XDR=extensively drug-resistant. XDR new drugs=standardised regimen plus Bedaquiline or delamanid.

For Romania, TB treatment features lengthy hospitalisation periods as listed in Table D.

**Table D. Average duration of hospital-focused and ambulatory TB treatment modalities for Romania from the 2017−2018 study [3]**

| **Treatment**  **regimen** | **Hospital-focused**  **(days)** | **Standard ambulatory**^1^  **(days)** |  |
| --- | --- | --- | --- |
| DS | 67 | 21 |  |
| MDR^2^ | 180 | 30−60 |  |
| XDR | 270 | 120−180 |  |

Treatment modalities include the use of directly observed treatment, short-course (DOTS), consisting of a standardised short-course anti-TB treatment given under direct and supportive observation

^1^Standard ambulatory care is the WHO recommended outpatient service delivery, with a reduced number of days hospitalised (hospitalisation only during the intensive phase of a given regimen or until smear conversion).

^2^MDR short-course consists of four month intensive phase (extended to six months in case of delayed sputum smear conversion) containing high-dose gatifloxacin or moxifloxacin, kanamycin, prothionamide, clofazimine, high-dose isoniazid, pyrazinamide and ethambutol followed by a continuation phase of five months containing gatifloxacin or moxifloxacin, clofazimine, ethambutol and pyrazinamide.

DS=drug-susceptible. MDR=multidrug-resistant. XDR=extensively drug-resistant.

For Romania, costs for all treatment programmes were estimated using a ‘bottom-up’ approach, based on average daily costs from hospital data as shown in Table E. An average cost per ambulatory interaction was also derived and applied to both screening programmes and to ambulatory treatment following the initial hospitalisation period. All programme costs were estimated by micro-costing using local data. For estimating treatment effectiveness, a quality factor of 0.8 was applied to account for the likely loss of impact between trial and real-world implementation [4].

# Table E. Component costs of TB treatment regimens for Romania from the 2017−2018 study (costs given in 2016 USD) [3]

| **Regimen** | **Hospital-focused costs** | **Ambulatory costs** | **Drug costs** | **Other costs** | **Total treatment cost** | **Diagnosis and monitoring costs** | **Total treatment cost with diagnosis** |
| --- | --- | --- | --- | --- | --- | --- | --- |
| Hospital-focused (DS) | 3,888 | 1,459 | 93 | 62 | 5,503 | 1,383 | 6,886 |
| Hospital-focused (MDR) | 10,386 | 3,287 | 5,256 | 145 | 19,074 | 5,212 | 24,286 |
| Hospital-focused (XDR) | 15,580 | 3,310 | 12,849 | 145 | 31,883 | 5,470 | 37,353 |
| Hospital-focused  (DS with incentives) | 3,888 | 1,459 | 93 | 211 | 5,653 | 1,383 | 7,036 |
| Hospital-focused  (MDR with incentives) | 10,386 | 3,287 | 5,256 | 387 | 19,316 | 5,212 | 24,528 |
| Hospital-focused  (MDR new drugs with incentives) | 10,386 | 3,287 | 6,541 | 298 | 20,512 | 5,212 | 25,724 |
| Hospital-focused  (XDR with incentives) | 15,580 | 3,310 | 12,849 | 388 | 32,126 | 5,470 | 37,596 |
| Hospital-focused  (XDR new drugs with incentives) | 15,580 | 3,310 | 29,202 | 439 | 48,531 | 5,470 | 54,001 |
| Ambulatory (DS) | 1,211 | 410 | 93 | 278 | 1,993 | 1,383 | 3,375 |
| Ambulatory (MDR) | 1,731 | 1,013 | 5,840 | 604 | 9,188 | 5,212 | 14,400 |
| Standard DOTS (DS) | 1,211 | 4,094 | 93 | 278 | 5,677 | 1,383 | 7,060 |
| Standard DOTS (MDR) | 1,731 | 10,128 | 5,840 | 604 | 18,302 | 5,212 | 23,514 |
| Standard DOTS (MDR new drugs) | 3,462 | 9,531 | 35,042 | 576 | 48,611 | 5,212 | 53,824 |
| Standard DOTS (XDR) | 6,924 | 10,178 | 5,840 | 606 | 23,549 | 5,470 | 29,019 |
| Standard DOTS (XDR – new drugs) | 10,386 | 8,986 | 35,042 | 551 | 54,965 | 5,470 | 60,435 |
| Prisoner DS | 3,888 | 1,459 | 93 | 62 | 5,503 | 1,383 | 6,886 |
| Prisoner MDR | 10,386 | 3,287 | 5,256 | - | 18,930 | 5,212 | 24,141 |
| Prisoner MDR new | 10,386 | 3,287 | 35,042 | - | 48,715 | 5,212 | 53,927 |

All programme costs were estimated by micro-costing using local data.

DOTS=directly observed treatment, short-course. DS=drug-susceptible. MDR=multidrug-resistant. New drugs=standardised regimen plus Bedaquiline or delamanid. With incentives=includes financial incentives. XDR=extensively drug-resistant.

| New active TB infections | | | | | | |
| --- | --- | --- | --- | --- | --- | --- |
|  | **Belarus [1]** | | **Moldova [2]** | | **Romania [3]** | |
| Years | Latest reported | Treatment optimised | Latest reported | Treatment optimised | Latest reported | Treatment optimised |
| 2015 | 3,569 | 3,569 | 5,253 | 5,253 | 16,644 | 16,644 |
| 2016 | 3,499 | 3,499 | 5,120 | 5,120 | 16,393 | 16,392 |
| 2017 | 3,433 | 3,433 | 5,074 | 5,074 | 16,159 | 16,159 |
| 2018 | 3,388 | 3,403 | 5,035 | 5,076 | 15,921 | 15,921 |
| 2019 | 3,342 | 3,296 | 4,995 | 5,109 | 15,681 | 15,681 |
| 2020 | 3,283 | 3,177 | 4,954 | 5,126 | 15,438 | 15,439 |
| 2021 | 3,222 | 3,075 | 4,913 | 5,095 | 15,194 | 15,194 |
| 2022 | 3,161 | 2,983 | 4,872 | 5,041 | 14,949 | 14,946 |
| 2023 | 3,101 | 2,902 | 4,832 | 4,983 | 14,703 | 14,696 |
| 2024 | 3,034 | 2,828 | 4,792 | 4,918 | 14,457 | 14,444 |
| 2025 | 2,959 | 2,760 | 4,753 | 4,846 | 14,211 | 14,189 |
| 2026 | 2,890 | 2,696 | 4,714 | 4,764 | 13,965 | 13,934 |
| 2027 | 2,830 | 2,637 | 4,677 | 4,676 | 13,721 | 13,677 |
| 2028 | 2,774 | 2,580 | 4,640 | 4,583 | 13,477 | 13,422 |
| 2029 | 2,723 | 2,526 | 4,604 | 4,484 | 13,235 | 13,168 |
| 2030 | 2,676 | 2,471 | 4,569 | 4,380 | 12,995 | 12,916 |
| 2031 | 2,630 | 2,416 | 4,535 | 4,274 | 12,756 | 12,665 |
| 2032 | 2,587 | 2,340 | 4,502 | 4,177 | 12,520 | 12,418 |
| 2033 | 2,545 | 2,299 | 4,470 | 4,087 | 12,285 | 12,172 |
| 2034 | 2,505 | 2,257 | 4,439 | 4,002 | 12,053 | 11,930 |
| 2035 | 2,466 | 2,215 | 4,409 | 3,921 | 11,824 | 11,690 |
| % change 2015 to 2035 | -31% | -38% | -16% | -25% | -29% | -30% |
| % change difference through optimisation 2015 to 2035 | Not  applicable | -7% | Not  applicable | -9% | Not  applicable | -1% |

Table F. Estimated number of new active TB infections under optimised allocation of treatment resources compared with latest reported for Belarus, Moldova, and Romania, 2015−2035

Table G. Estimated active TB prevalence per 100,000 under optimised allocation of treatment resources compared with latest reported for Belarus, Moldova, and Romania, 2015−2035

| **Active TB prevalence per 100,000** | | | | | | |
| --- | --- | --- | --- | --- | --- | --- |
|  | **Belarus [1]** | | **Moldova [2]** | | **Romania [3]** | |
| Years | **Latest reported** | **Treatment optimised** | **Latest reported** | **Treatment optimised** | **Latest reported** | **Treatment optimised** |
| 2015 | 131 | 131 | 296 | 296 | 129 | 129 |
| 2016 | 126 | 126 | 302 | 302 | 126 | 126 |
| 2017 | 121 | 121 | 304 | 304 | 124 | 124 |
| 2018 | 92 | 57 | 306 | 304 | 123 | 123 |
| 2019 | 68 | 34 | 306 | 303 | 121 | 121 |
| 2020 | 60 | 34 | 305 | 289 | 120 | 114 |
| 2021 | 56 | 32 | 304 | 271 | 118 | 108 |
| 2022 | 53 | 31 | 303 | 255 | 117 | 101 |
| 2023 | 47 | 30 | 301 | 240 | 116 | 94 |
| 2024 | 35 | 29 | 300 | 227 | 114 | 88 |
| 2025 | 33 | 29 | 298 | 215 | 113 | 81 |
| 2026 | 33 | 29 | 297 | 207 | 112 | 75 |
| 2027 | 33 | 28 | 295 | 199 | 110 | 73 |
| 2028 | 32 | 28 | 293 | 190 | 109 | 72 |
| 2029 | 32 | 26 | 292 | 181 | 108 | 71 |
| 2030 | 31 | 27 | 290 | 171 | 107 | 70 |
| 2031 | 31 | 26 | 289 | 166 | 105 | 69 |
| 2032 | 31 | 25 | 287 | 162 | 104 | 68 |
| 2033 | 30 | 24 | 286 | 158 | 103 | 67 |
| 2034 | 30 | 24 | 284 | 155 | 101 | 66 |
| 2035 | 30 | 23 | 283 | 151 | 100 | 65 |
| % change 2015 to 2035 | -77% | -83% | -4% | -49% | -23% | -50% |
| % change difference through optimisation 2015 to 2035 | Not  applicable | -5% | Not  applicable | -44% | Not  applicable | -27% |

Table H. Estimated TB-related deaths under optimised allocation of treatment resources compared with latest reported for Belarus, Moldova, and Romania, 2015−2035

| **TB-related deaths** | | | | | | |
| --- | --- | --- | --- | --- | --- | --- |
|  | **Belarus [1]** | | **Moldova [2]** | | **Romania [3]** | |
| Years | **Latest reported** | **Treatment optimised** | **Latest reported** | **Treatment optimised** | **Latest reported** | **Treatment optimised** |
| 2015 | 1,106 | 1,106 | 1,345 | 1,345 | 2,762 | 2,762 |
| 2016 | 1,064 | 1,064 | 1,378 | 1,378 | 2,694 | 2,694 |
| 2017 | 884 | 755 | 1,398 | 1,387 | 2,643 | 2,643 |
| 2018 | 770 | 367 | 1,410 | 1,385 | 2,598 | 2,599 |
| 2019 | 698 | 328 | 1,414 | 1,372 | 2,556 | 2,524 |
| 2020 | 633 | 316 | 1,414 | 1,317 | 2,515 | 2,395 |
| 2021 | 586 | 304 | 1,412 | 1,243 | 2,474 | 2,267 |
| 2022 | 523 | 296 | 1,409 | 1,166 | 2,434 | 2,141 |
| 2023 | 364 | 285 | 1,405 | 1,096 | 2,394 | 2,016 |
| 2024 | 318 | 279 | 1,400 | 1,032 | 2,353 | 1,892 |
| 2025 | 307 | 271 | 1,395 | 985 | 2,313 | 1,760 |
| 2026 | 303 | 265 | 1,391 | 953 | 2,273 | 1,633 |
| 2027 | 297 | 260 | 1,386 | 918 | 2,233 | 1,589 |
| 2028 | 292 | 253 | 1,381 | 881 | 2,194 | 1,558 |
| 2029 | 290 | 246 | 1,376 | 840 | 2,154 | 1,528 |
| 2030 | 285 | 240 | 1,372 | 799 | 2,115 | 1,497 |
| 2031 | 282 | 234 | 1,367 | 775 | 2,076 | 1,468 |
| 2032 | 277 | 224 | 1,363 | 756 | 2,037 | 1,439 |
| 2033 | 276 | 216 | 1,358 | 737 | 1,999 | 1,410 |
| 2034 | 272 | 212 | 1,354 | 718 | 1,961 | 1,382 |
| 2035 | 268 | 209 | 1,350 | 700 | 1,924 | 1,355 |
| % change 2015 to 2035 | -76% | -81% | 0% | -48% | -30% | -51% |
| % change difference through optimisation 2015 to 2035 | Not applicable | -5% | Not applicable | -48% | Not applicable | -21% |

**References**

1. Optimizing Investments in Belarus’ Tuberculosis Response. Washington DC: World Bank, 2017. Available from: [https://openknowledge.worldbank.org/bitstream/handle/10986/27475/116896.pdf?sequence=5&isAllowed=y.](https://openknowledge.worldbank.org/bitstream/handle/10986/27475/116896.pdf?sequence=5&isAllowed=y)

2. Optimizing Investments in Moldova's Tuberculosis Response. Washington DC: World Bank, 2018. Available from: <http://optimamodel.com/pubs/Moldova%202018.pdf>

3. Optimizing Investment in Romania's Tuberculosis Response Washington DC: World Bank, 2019. Available from: <https://openknowledge.worldbank.org/handle/10986/32594>

4. Watkins DA, Norheim OF, Jha P, Jamison DT. Reducing Mortality within Universal Health Coverage: The DCP3 Model. Seattle: DCP3, 2017. Available from: <http://dcp-3.org/resources/mortality-impact-achieving-essential-universal-health-coverage-low-and-middle-income>

**List of legends**

**Table A.** Duration of hospital-focused and ambulatory TB treatment by modality for Belarus from the 2016−2017 study [1]

**Table B.** Estimated cost of a full course of treatment for different drug regimens by treatment modality for Belarus from the 2016−2017 study (costs given in 2015 USD) [1]

**Table C.** Duration and cost of hospital-focused and ambulatory TB treatment modalities for Moldova from the 2017−2018 study (costs given in 2016 USD) [2]

**Table D.** Average duration of hospital-focused and ambulatory TB treatment modalities for Romania from the 2017−2018 study [3]

**Table E.** Component costs of TB treatment regimens for Romania from the 2017−2018 study (costs given in 2016 USD) [3]

**Table F.** Estimated number of new active TB infections under optimised allocation of treatment resources compared with latest reported for Belarus, Moldova, and Romania, 2015−2035

Table G. Estimated active TB prevalence per 100,000 under optimised allocation of treatment resources compared with latest reported for Belarus, Moldova, and Romania, 2015−2035

Table H. Estimated TB-related deaths under optimised allocation of treatment resources compared with latest reported for Belarus, Moldova, and Romania, 2015−2035
