# Supplementary material for: Public health benefits of shifting from hospital-focused to ambulatory TB care in Eastern Europe: Optimising TB investments in Belarus, the Republic of Moldova, and Romania
Source: PLOS Glob Public Health. 2023 Jun 21;3(6):e0001025. doi: 10.1371/journal.pgph.0001025 (PMC10284374; doi:10.1371/journal.pgph.0001025)
Supplement: Attachment — Submitted filename: TB EE response to reviewers.docx [file pgph.0001025.s002.docx]

Dear Dr Zwerling,

We appreciate the time and effort that you and the reviewers dedicated to providing feedback on our manuscript and are grateful for the insightful comments. We have incorporated most suggestions made by the reviewers, which have added value to our paper. Our response highlighting these changes have been noted below in blue and have been highlighted within our manuscript and the accompanying supplementary information.

Journal Requirements:

1. In order to meet journal requirements for reporting and reproducibility, at this time we request that you please update the Methods to report the original source of the data and the methods used to collect it in sufficient detail for another researcher to access the same data. In your Methods section, please include additional information about your dataset.

We have added a paragraph to the Methods section ‘Study data and costing’ listing the original sources of the data, providing additional information about these datasets and the methods used to collect these datasets, as well as pointing the readers to published reports with model inputs provided.

2. Please amend your detailed Financial Disclosure statement. This is published with the article. It must therefore be completed in full sentences and contain the exact wording you wish to be published.

a. Please clarify all sources of funding (financial or material support) for your study. List the grants (with grant number) or organizations (with url) that supported your study, including funding received from your institution.
b. State the initials, alongside each funding source, of each author to receive each grant.
c. State what role the funders took in the study. If the funders had no role in your study, please state: “The funders had no role in study design, data collection and analysis, decision to publish, or preparation of the manuscript.”
d. If any authors received a salary from any of your funders, please state which authors and which funders.

We thank the Editor for allowing us to revise the Funding Statement. The statement has been updated in the system and inserted on lines 628-630 of the manuscript. To note, no authors specifically received these funds, it is a trust so there is no grant number, and no author salaries were directly provided from this fund. Please let us know if you would like us to make any further changes.

The funding for this work was provided through a trust fund from the Joint United Nations Programme on HIV/AIDS (UNAIDS) ([https://www.unaids.org/](https://nam11.safelinks.protection.outlook.com/?url=https%3A%2F%2Fwww.unaids.org%2F&data=05%7C01%7Cncheikh%40worldbank.org%7C2815fb2dd914457121f308db325ba1ef%7C31a2fec0266b4c67b56e2796d8f59c36%7C0%7C0%7C638159142543092963%7CUnknown%7CTWFpbGZsb3d8eyJWIjoiMC4wLjAwMDAiLCJQIjoiV2luMzIiLCJBTiI6Ik1haWwiLCJXVCI6Mn0%3D%7C3000%7C%7C%7C&sdata=rfOLbefTPloG1u05d7A7OmKzmtuiLDwDd%2BFoE6zIyWA%3D&reserved=0)). The funders had no role in study design, data collection and analysis, decision to publish, or preparation of the manuscript.

Additional Editor Comments (if provided):
[Note: HTML markup is below. Please do not edit.]

Reviewers' comments:

Reviewer's Responses to Questions

**Comments to the Author**

1. Does this manuscript meet PLOS Global Public Health’s [publication criteria](https://journals.plos.org/globalpublichealth/s/criteria-for-publication)? Is the manuscript technically sound, and do the data support the conclusions? The manuscript must describe methodologically and ethically rigorous research with conclusions that are appropriately drawn based on the data presented.

Reviewer #1: Partly

Reviewer #2: Yes

2. Has the statistical analysis been performed appropriately and rigorously?

Reviewer #1: Yes

Reviewer #2: Yes

3. Have the authors made all data underlying the findings in their manuscript fully available (please refer to the Data Availability Statement at the start of the manuscript PDF file)?

The [PLOS Data policy](https://journals.plos.org/globalpublichealth/s/data-availability) requires authors to make all data underlying the findings described in their manuscript fully available without restriction, with rare exception. The data should be provided as part of the manuscript or its supporting information, or deposited to a public repository. For example, in addition to summary statistics, the data points behind means, medians and variance measures should be available. If there are restrictions on publicly sharing data—e.g. participant privacy or use of data from a third party—those must be specified.

Reviewer #1: No

Reviewer #2: No

We have added a paragraph to the Methods section ‘Study data and costing’ listing the original sources of the data, providing additional information about these datasets and the methods used to collect these datasets, as well as pointing the readers to published reports with model inputs provided.

4. Is the manuscript presented in an intelligible fashion and written in standard English?

PLOS Global Public Health does not copyedit accepted manuscripts, so the language in submitted articles must be clear, correct, and unambiguous. Any typographical or grammatical errors should be corrected at revision, so please note any specific errors here.

Reviewer #1: Yes

Reviewer #2: Yes

5. Review Comments to the Author

Please use the space provided to explain your answers to the questions above. You may also include additional comments for the author, including concerns about dual publication, research ethics, or publication ethics. (Please upload your review as an attachment if it exceeds 20,000 characters)

Reviewer #1: Comments to authors

Thank you for allowing me to review this manuscript, which reports on a modeling study of TB programs in Belarus, Moldova, and Romania. The authors demonstrate the benefit of transitioning from hospital-based TB care to ambulatory TB care. While the study design is interesting and yields valuable results, the additional benefit of this manuscript compared to the three World Bank country reports that were already published needs to be clarified. I also have additional comments detailed below which I hope are beneficial.

We thank the reviewer for their efforts in reviewing our manuscript and for their useful input. To demonstrate the additional benefit of this manuscript, we expanded lines 36-38 and 45-47 of the abstract and inserted an additional paragraph on lines 518-527 of the discussion.

General
1. From my understanding, this article is essentially a synthesis of 3 World Bank reports from 2017, 2018, and 2019. These reports, however, seem to have different (cost) data bases and different terminology of TB treatment regimens. The authors should try to merge terminology of TB treatments whenever possible (to allow the reader to compare between the 3 countries) and need to be more precise with respect to the cost data basis and assumptions for each country analysis.

We thank this reviewer for their comment. Where possible we have merged the terminology of TB treatments across counties to include DS (drug-susceptible); MDR (multidrug-resistant) standard and plus; pre-XDR (extensively drug-resistant) and XDR with or without new drugs as they apply to each country as shown in Supporting Information Table S2 for Moldova, Table S4 for Belarus, and Table S5 for Romania. We have made every effort to refine our precision of cost data basis; even reformatting to facilitate comparison across countries. To note, the currency reported for Belarus is US dollars (USD), whereas costs are reported in Euros for Moldova and Romania, based on country preference.

2. Additionally, it is not entirely clear to me what the additional benefit of this manuscript is compared to the three World Bank country analyses that were already published. The authors should state where they describe what is already published in those country reports and what the additional “original” content of the manuscript at hand is.

We have added a paragraph to the Methods section ‘Study data and costing’ listing the original sources of the data, providing additional information about these datasets and the methods used to collect these datasets, as well as pointing the readers to published reports with model inputs provided. To demonstrate the additional benefit of this manuscript, we expanded lines 36-38 and 45-47 of the abstract and inserted an additional paragraph on lines 517-524 of the discussion.

Abstract
1. The background section focuses on the epidemiology and costs of DR-TB. However, from my understanding, this does not directly relate to the study that the authors conducted, as the study’s focus is not DR-TB. The authors analyzed the distribution of resources between ambulatory and hospital-based DS-TB and DR-TB treatment. Hence, I would suggest that the authors motivate their study based on the question of ambulatory vs. hospital-based treatment, not on DR-TB treatment.

The basis of this study focuses on the higher cost of treating TB in hospital, versus through ambulatory care. Therefore transitioning from hospital-focused to ambulatory care will be cost saving, as well as will relieve the extra burden on the TB patient by reducing the length of stay in hospital, and will alleviate some of the strain on the healthcare system.

2. The findings section appears very brief (it is even shorter than the background section). It barely mentions concrete findings from the 3 countries. If word limit permits, I would suggest that the authors add more key findings to the abstract (e.g., concrete findings from the 3 countries).

We thank the reviewer for this helpful suggestion. We have now included key country-specific results in the Findings section of the Abstract, reporting the potential form reduced treatment cost by country from transitioning from hospital-focused care to ambulatory care.


Introduction
3. I found it hard to follow the reasoning of the introduction. In the first sentences, the authors mention that there has been a slow transition towards outpatient TB care in Eastern Europe. How does this causally relate to the high DR-TB rate in Eastern Europe mentioned in the second part of the first paragraph? Inpatient-centered TB care in FSU countries may have contributed to the rise of DR-TB, but it appears to be a multifactorial phenomenon. Thus, I suggest that the authors provide more info on inpatient care and rise of DR-TB in Eastern Europe/FSU countries in the intro (see, e.g., doi: 10.5588/ijtld.14.0190 or doi: 10.3855/jidc.3396). Additionally, I suggest you cite the most recent WHO global TB report.

We thank the reviewer for these comments. We have revised the introduction for better flow, have expanded the historical description of DR-TB in Eastern European/FSU countries, we cited Kohler et al. 2014 (10.5588/ijtld.14.0190), and in the first paragraph of the introduction, we have cited the 2022 WHO Global TB report.

4. I suggest you consider citing the systematic reviews by Fitzpatrick et al. 2012 (doi: 10.2165/11595340-000000000-00000) and Bassili et al. 2013 (doi: 10.4269/ajtmh.13-0004) when making the case for ambulatory care in the introduction.

Thank you, these reviews have now been cited in paragraph two of the introduction.

5. The first and third paragraph of the introduction start with the same sentences, and it seems they make very similar arguments. Consider re-writing the intro to have a coherent line of reasoning instead of reiterating similar arguments multiple times.

The repetitive sections have been removed. We thank this reviewer for this valid point.

6. In the fourth paragraph of the introduction, the authors describe a shift towards outpatient TB care during the Covid-19 pandemic. Please provide a reference for your claims.

This is a good point. The following reference (Martin-Hughes et al; [10.1371/journal.pgph.0000219](https://doi.org/10.1371/journal.pgph.0000219)) has been provided on lines 122 and 535.

7. The introduction focuses on DR-TB treatment, but DS-TB is also part of the modeling. I suggest the authors also include WHO recommendations for ambulatory DS-TB treatment in the introduction.

We thank the reviewer for this comment, and have amended to include the WHO recommendation for DS-TB below table 2 (footnote one), as we did not find a suitable section in the introduction.

Methods
8. Table 2 summarizes the outpatient-based treatments for Belarus, Moldova, and Romania. Although I understand that there are differences in TB drug regimens and terminology between the countries, I struggle understanding the table: For instance, what does “Considered” mean compared to “standard and incentivized”? What is “incentivized”? For MDR-TB, what is “MDR classic” and “MDR plus”, and how does this compare to “MDR standard”? What do the authors understand as short-course MDR-TB treatment (with injectables or without injectables or the BPaL(M) regimen)? I think additional explanations and definitions on the terminology of drug regimens mentioned in the table would aid understanding. Additionally, the authors could standardize the terminology across countries (referring to the terminology of WHO-recommended treatments). This would help synthesize the separate analyses of the three countries.

Terms have been standardized across countries following WHO-recommended and defined in Table 2 and in the Supplement.

9. The Outcomes section of the article stipulates that ambulatory treatment is superior compared to hospital-based treatment. This already constitutes results/interpretations instead of methodology. In the “Outcomes” paragraph, one would expect an explanation of the outcome variables of interest.

We have reworded the ‘Outcomes’ section of the Materials and Methods to avoid making a results-based conclusion, but instead now state that less costly, but equally effective (following findings reported in the WHO guidelines) ambulatory DR-TB would be prioritised by the model. The financial and health outcomes have also been further clarified.

10. According to the methodology, the authors performed an optimization of TB care spending for each country, which was not limited to the question of investing in hospital-based or ambulatory care, but also included other treatment interventions (e.g., palliative care, prison-based treatment). Did the authors pre-define that an “optimized” scenario had to include mostly ambulatory care (and thereby pre-defined some parameters of the optimized solution), or did ambulatory care simply come up as the optimal solution for each country? This comment also touches the study motivation: Did the authors conduct a study to determine the potential benefits of ambulatory over hospital-based care, or did the authors conduct a study to optimize TB care in the respective countries? At the moment, these two distinct study motivations are mixed throughout the manuscript.

We thank the reviewer for this overarching comment. We have accordingly clarified throughout. To highlight as previously stated starting on line 179 of the manuscript “The objective for these published studies was to identify the most cost-effective resource allocation across existing and prospective TB diagnosis and treatment modalities to minimise a combination of active TB cases, prevalence of active TB, and TB-related deaths by 2035. A primary focus was to determine the health benefits and savings that could be gained by shifting from hospital-focused to ambulatory TB care.” The optimisation did not prescribe the shift to ambulatory TB care as a model input, this was an outcome of each study. However, with the available evidence from each country being that ambulatory care has a lower cost per patient treated and identical (or improved with incentives) treatment outcomes for patients where additional inpatient care is not medically advised (supporting evidence with references have been reinforced), this was also an inevitable recommendation from early in each study. Accordingly, the focus of each optimisation analysis was how to optimally allocate those potential cost-savings (by reinvesting in higher-impact interventions, as described in our manuscript), and the epidemiological and public benefit of doing so. This has also been further clarified in the abstract and in the introduction, as described in responses to the above comments kindly provided by the reviewer.

11. Most likely, despite shifts towards ambulatory care, some TB patients will still require hospitalization due to their severity of illness or comorbidities. How was this factored in the model?

Indeed this was modelled, we thank the reviewer for pointing out that this was not clear in our paper. We have clarified that all modalities are represented by an average number of days of hospitalization (as shown in Appendix Tables S1, S3, and S4), which account for some portion of people clinically requiring longer hospitalization.

In addition, each country applied different maximum constraints on the proportion of people with TB who would be eligible for a shift to ambulatory care. In Belarus a constraint was applied that at least 30% of most recently reported spending on hospital-focused treatment for all TB strains would continue, and 20% of involuntary isolation. In Moldova the constraint was at least 10% for DS-TB and MDR-TB and 15% for XDR-TB. In Romania no constraint was used for hospital-focused care but a minimum-constraint on DOTS of 40% of TB patients was applied. This has been noted on lines 295-304.

12. As the reports for the 3 countries stem from different years, how did the authors standardize costs to the same year and currency? What was the currency year used in the analysis?

The focus of this analysis is not to directly compare country costs, but rather the relative reduction in costs through a shift to ambulatory care and optimisation of resources including the availability of increased ambulatory care, so we do not consider that currency standardisation is necessary to interpret the conclusions. The Belarus costings were reported in 2015 USD, Moldova and Romania each used 2016 EUR as the baseline year. This has now being fully clarified in the methods and caption for figure 1, where annual allocations are illustrated.

13. What was the perspective of the cost analyses in the 3 countries? Were costs determined from a societal perspective or health system perspective? What was the time horizon of cost collection? What was the discount rate? This basic data of economic evaluations needs to be included in the methods section.

Thank you for this comment, costs were determined through bottom-up costing exercises that were carried out by country teams for the most recent 2-3 full years of available cost data for each country. This costing captures health system costs and not any additional societal costs. As ambulatory care by design incurs less societal costs (e.g. less time in hospital means fewer days of lost workplace productivity), capturing all societal costs would only strengthen these findings.

We have clarified that no discounting was applied to costs in the ‘Study data and costing’ section of the methods. To note that while discounting would be sensible for future cumulative projections of costs relative to future cumulative projections of impact, we did not apply discounting as our objective was not to draw these types of comparisons. For the optimisation analyses, since we do not consider individual cost-effectiveness estimates (e.g. ICERs), we do not believe it would be useful to present discounted outcomes (e.g. discounted future deaths).

Results
14. Minor: Please add a y-axis label in Figure 2.

A y-axis label has been added to Figure 2.

15. Figure 2: What do the authors mean by “TB treatment coverage”? Is it the number of people treated for the respective TB type? And do the optimization bars refer to the year 2035? Do the optimized scenarios include re-investment of saved resources in TB care?

In the manuscript we recommend that “Immediate savings from transitioning from involuntary isolation and other hospital-focused treatment to ambulatory care, as well as savings if new cases decline as projected meaning reduced need for treatment, should be reallocated to higher impact program interventions and delivery solutions”, but also show that “if resources for TB were optimally reallocated from 2015 to 2035 for Belarus, Moldova, and Romania, including prioritising less expensive but equally effective ambulatory TB care (therefore more cost-effective) over hospital-focused care, and assuming these savings remained in the TB budget and were optimally reinvested across TB interventions, then new active TB infections could be reduced by 9% in Moldova (1% in Romania and 7% in Belarus), active TB prevalence per 100,000 reduced by 44% in Moldova (5% in Belarus and 27% in Romania), and TB-related deaths reduced by 48% in Moldova (5% in Belarus and 21% in Romania) over this period (Fig 3 with corresponding estimates reported in Supporting Information Tables S6−S8).“

16. Figure 3: Do the projections of TB prevalence, active TB cases, and TB-related deaths until 2035 include a re-investment of resources that were freed up by re-allocation of resources?

Yes, the optimized allocations include re-investment of resources that were freed up by re-allocation.

17. General note: I suggest the authors revise the results to clearly reflect the time dimension of each of their results. I found it difficult to understand whether the authors discuss potential savings that can be achieved by redistribution at the initial year of analysis or savings projections until 2035, and whether these include a re-investment of freed resources in other TB interventions.

We have clarified where applicable. To note, figure 1, annual optimisations are not year dependent, we revised the figure caption to clarify. We have also noted that the optimised budget excluded reinvestment of freed resources from other TB interventions.

18. For some TB resistance types, Table 1 stipulates more than one treatment option. For example, DS-TB treatment in Belarus may be “standard” or “incentivized”, or long-course MDR-TB treatment in Moldova may be “MDR classic” or “MDR plus”. The results, however, only discuss the general distribution of resources among TB resistance types and ambulatory vs. hospital-based care. How were resources distributed between different treatment options within the same TB resistance type?

Terms have been standardized across countries following WHO-recommended and defined in Table 2 and in the Supplement.

19. Consider adding subheadings (maybe one subheading for each country) to the results section.

This is a useful suggestion, thank you. Subheadings have been added to the results section.

Discussion
20. Meta-analyses (some of which you cite) indicated that decentralized care has similar or even better treatment outcomes than hospital-based care, but this should be taken with a grain of salt. Evidence stems from cohort studies (some with historical treatment groups), and there are no randomized trials at hand. Existing studies are therefore prone to selection bias, where sicker patients tend to receive hospital-based treatment (and tend to have worse outcomes), and patients who are not so ill are treated ambulatorily (with better outcomes). This argument is worth mentioning in the discussion.

This is a good point, and has been described in the second paragraph of the introduction.

21. As mentioned in a previous comment on the introduction, the authors need to provide references in the paragraph on Covid-19 to back up their statements on transitions to ambulatory care that took place during the Covid-19 pandemic.

Same as above, the following reference (Martin-Hughes et al; [10.1371/journal.pgph.0000219](https://doi.org/10.1371/journal.pgph.0000219)) has been provided on lines 122 and 535.

22. On a similar note, is there evidence for the following 4 claims the authors make in the discussion? “Other benefits include reduced nosocomial transmission-related health systems costs, cost (direct and indirect) to the patient, as well as reduction in infection risk, and stigma surrounding access to longer-term hospital care.”

This sentence has been simplified as follows “Other benefits include reduced nosocomial transmission-related health systems costs (13) and cost (direct and indirect) to the patient ().” Reduction of nosocomial risk and associated costs have been supported with an additional reference ([10.3855/jidc.3396](https://doi.org/10.3855/jidc.3396)). And patient costs have been supported with an additional reference ([10.1007/s40273-015-0279-6](https://doi.org/10.1007%2Fs40273-015-0279-6)).

23. With results from the Nix, ZeNix, and TB PRACTECAL trials, recommended DR-TB treatment has been changing dramatically over the last 5 years. Changing DR-TB drug regimens and treatment durations appear to be a major limitation of findings of the modeling that need to be addressed in the discussion.

While we acknowledge that DR-TB drug regimens and costs have quantitatively changed since these three original studies were completed, however, our recommendations to transition to ambulatory care to save costs remain relevant. This statement has been added on lines 590-593, and follow the limitation handling direct cost comparisons.

24. The concluding paragraph seems to be unrelated to the results of the study. Ideally, this paragraph should reflect key results of the study in conjunction with central interpretations/recommendations.

We agree with the reviewer and have revised the last paragraph of the discussion accordingly.

Reviewer #2: Dear authors,
Thank you for submitting the paper titled “Public health benefits of shifting from inpatient to outpatient TB care in Eastern Europe: optimising TB investments in Belarus, the Republic of Moldova, and Romania” for potential publication in PLOS Global Public Health as a Research Article. The topic is interesting, and the use of the relatively new tool Optima TB may reveal some new lessons about the adoption of outpatient DR-TB care in the region. I believe it can be published if some minor revisions are made to the current version of the paper.

We thank the reviewer for their efforts in reviewing our manuscript and for their kind comments, and useful points.

Introduction:
1. At four pages, including a table, I find the introduction to be unduly long. Authors should reduce this section by summarising more or moving parts of this to the discussion section.

This has been addressed by removing the duplication and revising for flow to address the comment from reviewer #1 (noting that reviewer #1 asked us to provide addition historical details).

2. Line 50 attributes the increases in rates of new cases of MDR/RR to the inpatient care system in place. Can they provide references to support this?

To address comment #1, this sentence has been removed.

3. The last phrase in line 87 is a repetition of what was state in line 85.

We have revised this section to remove repetition.

4. The sentences starting in line 104 and ending line 107 can be moved to methods.

These sentences have been shortened to remove repetition and moved to the ‘Outcomes’ section under public health impact outcomes

5. It is not exactly clear what ‘these studies’ in line 109 refer to – is it the current study or the cited studies?

We have clarified that this statement refers to the original these country studies (as published in the three country reports). These studies are examined with collective findings revealed in this study.

6. Lines 109 to 124 could be moved to discussion.

We agree. These two paragraphs have been moved to lines 487-502 of the discussion.

Methods:
7. Author name and date missing in line 129 before (15) to complete the sentence.

This has been revised accordingly (line 114).

8. Even though the authors reference in the (15) reference, a brief description of Optima TB will be helpful here – including how it was developed and validated and if it has been used successfully in similar contexts.

##### This is useful. We have provided a brief description on lines 172-174 of the materials and methods.

9. The sentence starting on line 130 on stakeholders is out of place in this section and should be moved to the ‘Study data and costing’.

We agree. This sentence has been removed, as it has now already been captured in the ‘Study data and costing’.

Results:
10. I believe this section will be clearer if reorganised. I propose discussing each country separately before proceeding to how they compare. Although the authors have attempted to do that, there are sections discussing the 3 countries in between the texts for each country, which is a bit distracting. Eg, after beginning with Belarus, the authors mention Moldova and Romania in lines 250 to 251, then return to Belarus in lines 251 before going on to talk about Moldova in lines 264-280 and very briefly to Romania in 282 to 286. Authors could move the Moldova and Romania mention on line 250 to after line 286.

We thank the reviewer for their careful review and detailing. This revision has been made accordingly. The sentence “Under optimised allocation, treatment coverage in Moldova and Romania would surpass the most recently reported (at the time of analysis) need, 121% and 104%, respectively”, now appears on lines 426-427 of the results.

Discussions:
11. Can you discuss how your study compares to similar modeling studies in similar contexts?

These studies have been compared and contrasted with each other. Other Optima TB modelling studies have been conducted, for example for countries in sub-Saharan Africa, however, the context and objectives were different, and thus they cannot be readily compared. We have now referenced similar results from a study conducted in Bulgaria (Doan et al. Strategic investment in tuberculosis control in the Republic of Bulgaria. Epidemiology & Infection, 2019, doi [10.1017/S0950268819001857](https://doi.org/10.1017/s0950268819001857)) on lines 526-527.

12. Please add a paragraph on the limitations of your approach and findings.

A limitations paragraph has been added on lines 569-593 of the discussion.

6. PLOS authors have the option to publish the peer review history of their article ([what does this mean?](https://journals.plos.org/globalpublichealth/s/editorial-and-peer-review-process#loc-peer-review-history)). If published, this will include your full peer review and any attached files.


**Do you want your identity to be public for this peer review?** If you choose “no”, your identity will remain anonymous but your review may still be made public.


For information about this choice, including consent withdrawal, please see our [Privacy Policy](https://www.plos.org/privacy-policy).

Reviewer #1: **Yes:** Nicolas Paul

Reviewer #2: No
